# Dynamic Inference Approach Based on Rules Engine in Intelligent Edge Computing for Building Environment Control

**DOI:** 10.3390/s21020630

**Published:** 2021-01-18

**Authors:** Wenquan Jin, Rongxu Xu, Sunhwan Lim, Dong-Hwan Park, Chanwon Park, Dohyeun Kim

**Affiliations:** 1 Big Data Research Center, Jeju National University, Jeju 63243, Korea; wenquan.jin@jejunu.ac.kr; 2 Department of Computer Engineering, Jeju National University, Jeju 63243, Korea; rongxu@jejunu.ac.kr; 3 Autonomous IoT Research Section/Intelligent Convergence Research Laboratory, Electronics and Telecommunications Research Institute, Daejeon 34129, Korea; shlim@etri.re.kr (S.L.); dhpark@etri.re.kr (D.-H.P.); cwp@etri.re.kr (C.P.)

**Keywords:** edge computing, deep learning, rules engine, inference model, computational offloading

## Abstract

Computation offloading enables intensive computational tasks in edge computing to be separated into multiple computing resources of the server to overcome hardware limitations. Deep learning derives the inference approach based on the learning approach with a volume of data using a sufficient computing resource. However, deploying the domain-specific inference approaches to edge computing provides intelligent services close to the edge of the networks. In this paper, we propose intelligent edge computing by providing a dynamic inference approach for building environment control. The dynamic inference approach is provided based on the rules engine that is deployed on the edge gateway to select an inference function by the triggered rule. The edge gateway is deployed in the entry of a network edge and provides comprehensive functions, including device management, device proxy, client service, intelligent service and rules engine. The functions are provided by microservices provider modules that enable flexibility, extensibility and light weight for offloading domain-specific solutions to the edge gateway. Additionally, the intelligent services can be updated through offloading the microservices provider module with the inference models. Then, using the rules engine, the edge gateway operates an intelligent scenario based on the deployed rule profile by requesting the inference model of the intelligent service provider. The inference models are derived by training the building user data with the deep learning model using the edge server, which provides a high-performance computing resource. The intelligent service provider includes inference models and provides intelligent functions in the edge gateway using a constrained hardware resource based on microservices. Moreover, for bridging the Internet of Things (IoT) device network to the Internet, the gateway provides device management and proxy to enable device access to web clients.

## 1. Introduction

Edge computing brings resources of computation and storage to the edge of networks for providing various functions to the Internet of Things (IoT) devices with sensors and actuators. Based on edge computing, the constrained IoT network is supported to operate heterogeneous solutions, such as management, intelligent approaches, proxies and autonomous control mechanisms, to provide rich service scenarios [1,2,3,4,5]. Due to IoT networks deploying constrained devices for sensing and actuating, based on the limited power supply, processor, storage, and network capability, this environment struggles to support a sufficient computation and network requirement for implementing complex service scenarios [6,7,8]. For overcoming the limitations of IoT, cloud computing can be a solution to provide effectively scalable and easily accessible interfaces to IoT networks as well as web clients [9]. Nevertheless, edge computing enables the computing resources to be performed close to the environment where the data are generated and applied by sensors and actuators [10,11]. According to the edge computing architecture, edge computing deploys the device to serve in the middle layer between IoT devices and the cloud elements.

The edge gateway is a device that provides edge computing functions to interact with IoT devices and cloud elements from the entry of the network edge [12,13,14]. The edge gateway provides management functionality through monitoring the environment and devices, registering the information of devices for discovery by web clients and forwarding messages to different protocols and networks [15,16,17,18]. Additionally, the intelligent services are provided based on the sufficient computational ability of the edge gateway [19]. Therefore, the edge gateway enables multiple solutions to be deployed on the edge of networks for providing the management, proxy, intelligent and autonomous services close to the environments. For integrating multiple solutions to the edge gateway to provide comprehensive services, the microservices architecture is key. Using the microservices provider modules in the edge gateway, the deployment of services is enabled to be flexible, lightweight and extendable [20,21,22,23,24]. Based on the independent microservice providers, the services can be provided to external service consumers for a specific purpose as well as to internal functions for the collaboration mechanisms. Moreover, the deployment of multiple intelligent approaches is enabled without updating other functions to apply intelligent solutions dynamically.

The computational offloading leverages multiple computational resources to separate intensive computational tasks for deploying light-weight tasks in the network edge to reduce computing load and network latency. Deep learning is an approach to develop intelligent services based on a volume of sample data, which results in a model of prediction or decision [25]. For providing intelligent services based on deep learning, firstly, the learning model is trained with the data to derive the inference model. Then, the service provider includes the inference model to provide intelligent services with the required input parameters. By updating training data to extend the knowledge of the existing learning model, it is possible to use the environment data in the edge gateway [26,27]. However, the training process takes time, especially in the edge gateway. Therefore, training the learning models with various datasets in the cloud server and deploying the inference modes in the edge gateway enables the operation of multiple intelligent services on the edge of networks.

This paper proposes a dynamic inference approach through deploying an intelligent function and rules engine in the edge gateway for providing intelligent edge computing in the building environment. Intelligent edge computing is enabled based on the edge gateway that is deployed in the entry of the network edge to provide services for device management, device proxy, client service, intelligent service and rules engine using microservice providers. The intelligent function interprets the inference models using the model interpreter, which in turn provides the intelligent services that are used by the rules engine in the edge gateway. The rules engine provides the dynamic inference approach based on the rules that are used to simulate various intelligent service scenarios in the IoT device networks. Using the proposed intelligent edge computing in the building environment, the edge gateway delivers the energy consumption value to the IoT device, which updates the environment parameters, including temperature and humidity, by controlling the heater. For registering the IoT devices to represent in the cyber world, a device management technique is developed based on the EdgeX framework [28], which provides various management interfaces through microservices. Additionally, a client service provider and device proxy are developed and deployed in the edge gateway to connect the Internet to the IoT device network. We performed an experiment to evaluate the performance of the proposed intelligent edge computing through comparing with the external intelligent function. The experimental results show that the proposed edge gateway takes less time to deliver the control factor to the environment.

The rest of this paper is organized as follows. Section 2 introduces the related works regarding edge computing frameworks and solutions for enabling the intelligent approaches in the edge of networks based on computational offloading and embedding. Section 3 presents the proposed intelligent edge computing architecture and functional blocks. Section 4 introduces the proposed dynamic inference approach and related solutions, including computational offloading and rules engine. Section 5 presents the implementation details and results of the IoT device, edge gateway and edge server. Section 6 evaluates the performance of the edge gateway with the proposed microservices provider modules. Finally, we conclude this paper and introduce our future directions in Section 7.

## 2. Related Works 

Edge computing provides computational resources close to the environment for reducing the computational load in the IoT networks and latency. EdgeX framework [28] is an emerging edge computing solution that comprises multiple microservice provider modules to provide management of devices and data, and communications for heterogeneous devices. The microservices enable the scale of functionality to be up and down based on the capability of the edge gateway [29]. It does this by deploying various domain-specific service providers based on microservices, which enables providing improved services in edge computing. Mobile edge computing (MEC) is proposed to enable data service and cloud computing tasks on the edge of networks that provide a sufficient computing ability based on a high-performance device [30,31,32]. The architecture of MEC provides computational offloading for distributing the computational load to multiple mobile devices [33]. Cloudlet [34] comprises multiple computing devices and abundant resources of the network, computing and storage to provide trusted support to nearby mobile devices for enabling various scenarios based on mobile-cloud convergence [35,36]. MEC and Cloudlet are used for implementing cloud computing in mobile devices to enable computing and data repositories on the edge of the networks [37,38,39,40,41]. However, the limitation of the mobile device only supports a small-scale of computational resources. Offloading the heavy tasks to the cloud server can overcome the limitation and reduce the computational load for the network edge.

Most task offloading approaches are used for deploying deep learning models with huge volumes of data to the high-performance computing unit [42]. Eom et al. [43] proposed a mobile offloading framework to apply machine learning techniques based on an adaptive scheduling approach. Qiao et al. [44] proposed an integrated task offloading to efficiently combine the services from the multiple mobile service providers in MEC for vehicular networks. Xu et al. [45] proposed intelligent edge computing based on offloading the learning approach to the cloud for renewable power supply. Crutcher et al. [46] proposed a task offloading mechanism to deploy the learning model to the cloud for reducing the resource consumption based on a utilized prediction approach. Kwak et al. [47] proposed an energy optimization approach through offloading the heavy computing process to the cloud server for minimizing the energy in private edge computing. However, deep learning approaches need time to train the model with huge volumes of data, and only some computing units have high enough computational resources while still being able to fit in a small-sized device. Zhang et al. [48] proposed a real-time object detection technique using NVIDIA Jetson TX1, which is an embedded device that presents sufficient performance. Beatriz et al. [49] developed a multiple object visual tracking based on implementing real-time deep learning on the NVIDIA Jetson TX2 development kit, which provides a powerful deep learning computing processor through a constrained mobile device. Nevertheless, operating the deep learning model in constrained devices requires a high cost for purchasing the computing units. Therefore, offloading the learning model to the cloud server and deploying the inference model on the network edge is a better option.

## 3. Intelligent Edge Computing for Building Environment Control

**Figure 1:**
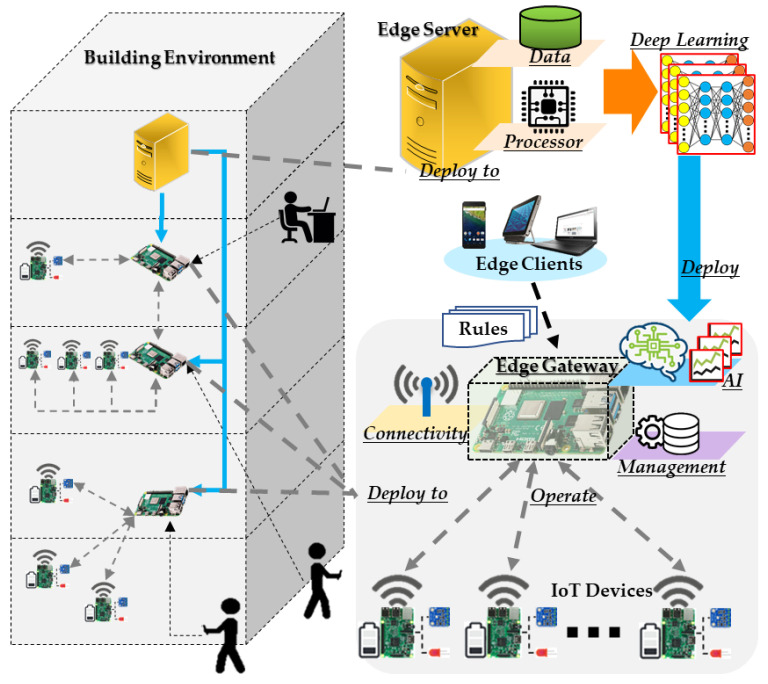
Proposed intelligent edge computing for building environment control.

We propose an intelligent edge computing for building environment control. As shown in Figure 1, the proposed edge computing comprises an edge server, edge gateway, edge client and IoT device. For deploying intelligent edge computing in the building environment, the edge gateway provides intelligent approaches through offloading the inference model to the building environment. In the building environment, IoT devices are deployed to provide sensing and actuating services. The IoT devices are connected to a network, which can be a local network that serves a private space in the building. The edge gateway is deployed in the entry of the local network to interact with the IoT devices. For enhancing the ability of the IoT devices in the network, the edge gateway provides management, connectivity and intelligent solutions through sufficient computational resources. The IoT devices are connected to the edge gateway; therefore, the edge gateway collects the sensing data and controls the actuators through communication with the IoT devices. Additionally, from the Internet, the edge client accesses the IoT devices through the edge gateway. 

For providing the intelligence in the building environment, the deep learning models are offloaded to the edge server, which derives the intelligent models based on high-performance computational resources and a huge volume of building user data. Then, the intelligent models are deployed to the edge gateway for offloading intelligent approaches to the network edge for operating intelligent scenarios using IoT devices in the building environment. Based on the rules, offloaded intelligent approaches are selected to apply to the building environment for specific scenarios. 

**Figure 2:**
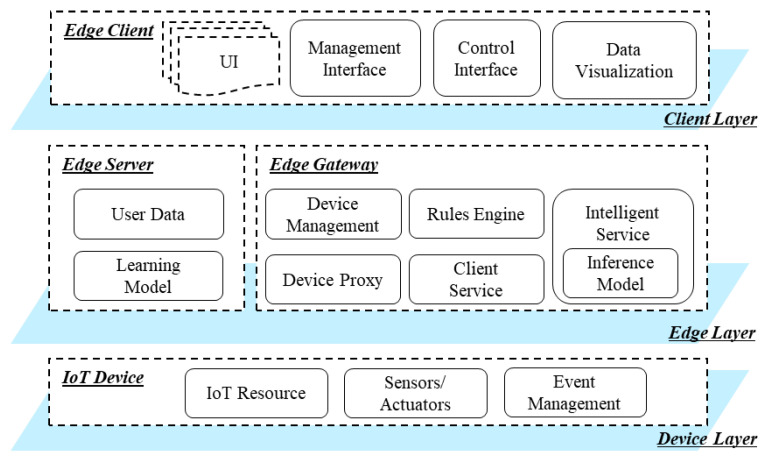
Intelligent edge computing hierarchical architecture.

The functions of the proposed edge computing are separated into three layers, including client, edge and device layers, as shown in Figure 2. 

The device layer includes IoT devices that are used for collecting data from the environment and update the environmental parameters through controlling actuators. The IoT device equips with sensors and actuators that are represented in the IoT device by resources. The resources expose the functions of sensing and actuating in the network for providing the IoT services to the edge gateway. The resources are developed based on IoT frameworks and libraries that enable the IoT device to provides services for delivering the data to the network elements. The IoT device includes the wireless or wired connection ability to communicate with the edge gateway through network protocols such as the Constrained Application Protocol (CoAP) and Hypertext Transfer Protocol (HTTP). Event management is used for publishing the sensing data and device status to the edge gateway, which enables the collection of continuous data from the environment and detects the real-time event. 

The edge layer includes edge server and edge gateway to distribute computing operating IoT devices based on intelligent approaches in the edge of networks. The edge server involves user data and learning models to result in the inference model through training the user data on the learning model. The deep learning models are designed to generate a network that outputs the results by passing the inputs between nodes. The links between nodes of the network are defined by updating the parameters of the links based on a large amount of data. Therefore, a heavy computing process is required, which is supported by the edge server. The inference model is deployed in the edge gateway for deriving a smart result to control the IoT device. For interacting with IoT devices, the edge gateway provides management and device proxy services to link the IoT devices to the cyber world. Moreover, the client service provider enables the users to access the represented virtual object to access actual devices through the Internet.

The client layer includes the edge client that is used for providing content to the users and sending the command to the system by interacting with users and the edge gateway. Based on the edge client, the client layer involves functions of User Interface (UI), management interface, control interface and data visualization. The UIs are provided by the edge gateway to the edge client, and displayed to the users. Using the UIs, management services, control interfaces and data visualization are provided, such as writing and reading device information, configuring system parameters, sending the command to IoT device and presenting sensing data in various styles. The client layer is required for any system that interacts with the server to present the contents of the services.

## 4. Proposed Dynamic Inference Approach Based on Rules Engine

**Figure 3:**
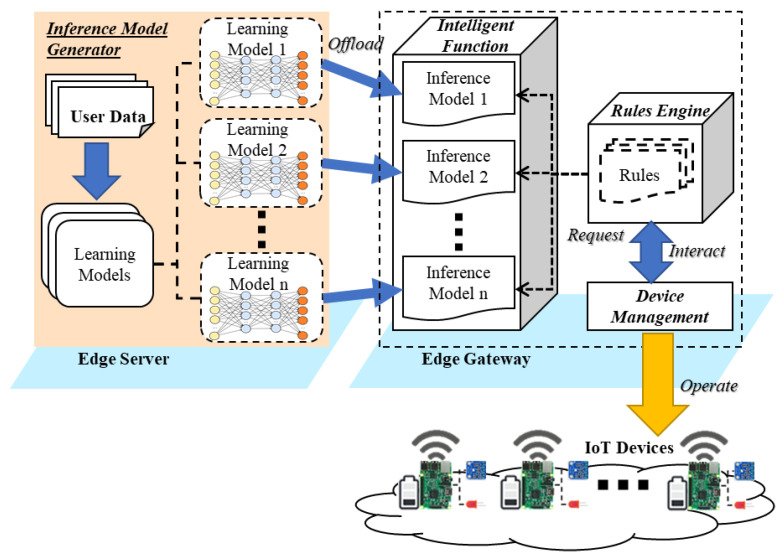
Offloading flow for embedding the inference model to the rule-based edge gateway.

In this section, we present the dynamic inference approach that is provided by the rules engine based on interacting with multiple inference models. Figure 3 shows the offloading flow for embedding the inference model to the edge gateway. In the edge server, the inference model generator derives the learning models by applying the building user data to the learning models. Each learning model outputs an inference model that is offloaded to the edge gateway to provide intelligent service. The inference models are deployed in the intelligent service provider, which includes intelligent functions to provide building environment control factors based on the required input parameters. The intelligent functions are invoked by the rules engine based on the rules that define the IoT device operation scenarios. According to the rule profile, the rules engine applies the intelligent approach dynamically. The intelligent function provides the control factor based on the inference model and delivers the control factor to the device network for operating the actuator to update the building environment. 

**Figure 4:**
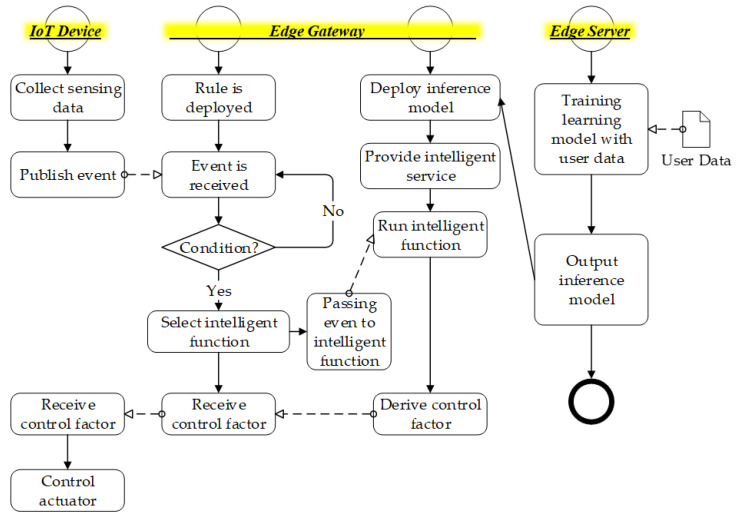
Deployment flow of embedding inference model to rule-based edge gateway.

As shown in Figure 4, the IoT device publishes the event to the edge gateway and the rules engine operated the scenario base on the intelligent function. The intelligent function is triggered by the event that is sent from the IoT device. The event is a request message to include sensing data and actuating commands. The rules engine makes the decision to operate an intelligent scenario. Then, the rules engine delivers the event to the selected intelligent function that is provided based on the inference model. The inference model is derived from the deep learning that is developed based on user data.

**Figure 5:**
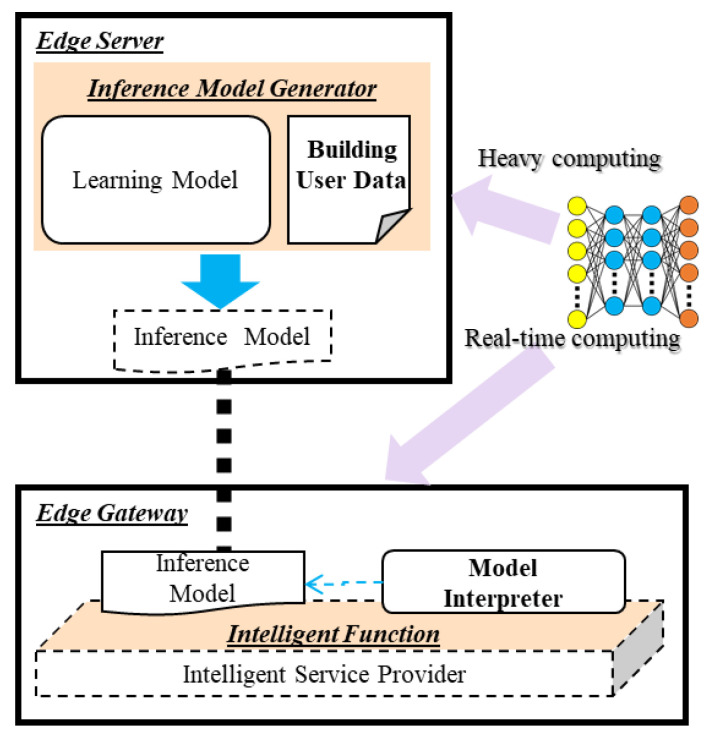
Scenario of the offloading learning and inference model for intelligent edge computing.

Figure 5 shows the scenario of the offloading learning and inference model to distributed edge computing. The computational process of deep learning is distributed to the edge server and edge gateway. The edge server includes the inference model generator to provide the inference model that is used in the edge gateway. For generating the inference model, the inference model generator includes the learning model and building user data, and trains the learning model using the data. Then, the edge gateway deploys the inference model on the intelligent service provider to provide intelligent service. The intelligent service provider includes the intelligent function that interprets the inference model using the model interpreter for providing the intelligent service based on the inference model.

**Figure 6:**
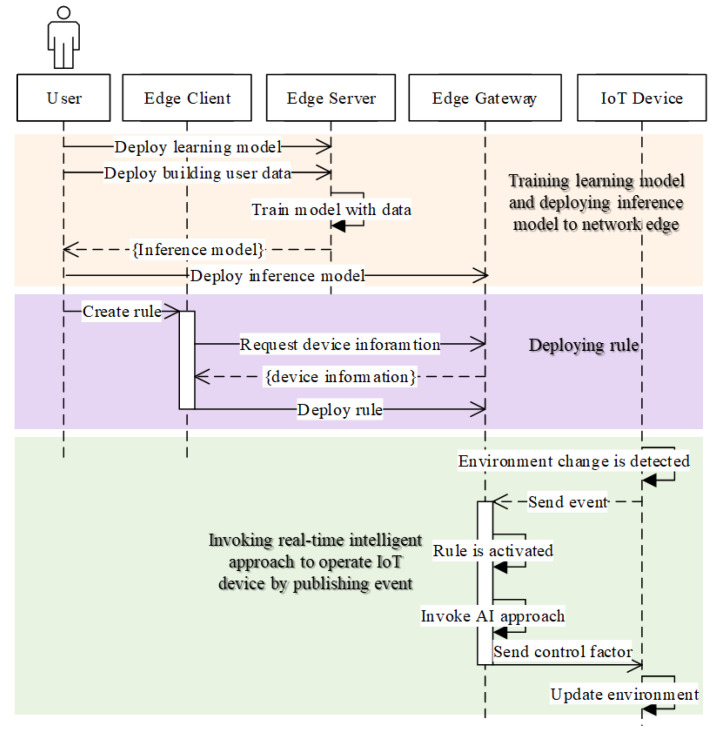
Intelligent IoT device operation sequence.

Figure 6 shows the intelligent IoT device operation sequence. The sequence presents the process of training the learning model and deploying the inference model to the network edge. Once the inference model is deployed, the user deploys the rule through the edge client and the rule engine requests to the real-time intelligent service provider to operate IoT devices by publishing events. First, the user deploys the learning model and building user data, and trains the model with the data to get the inference model. The model will be deployed in the edge gateway. Second, the user crates the rule using the edge client through requesting device information and deploying the rule to the edge gateway. Finally, the edge gateway activates the rule to invoke the intelligent service provider and control the IoT device.

## 5. Implementation Details and Results

**Figure 7:**
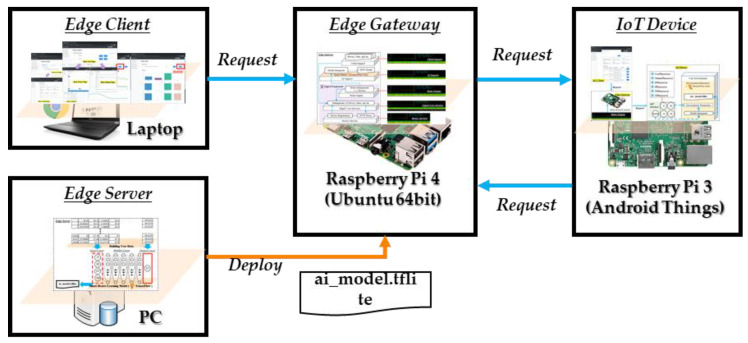
Implemented entities of the proposed embedded edge computing.

The proposed entities are deployed in the corresponding platform to provide functions as shown in Figure 7. The edge client is a web client that provides the information through UIs to users as well as receiving the information from users and delivering it to the edge gateway. In the experiment, the Chrome web browser runs the edge client on a laptop to access the edge gateway through the Internet. The UIs are provided by the client service provider from the edge gateway. Bootstrap and jQuery libraries are used for implementing the contents of UIs based on the Spring Boot framework. 

The edge gateway includes modules of the client service provider, intelligent service provider, EdgeX core, rules engine and device proxy for providing services to edge client and IoT device. Spring Boot 2.1.4 is used for developing the client service provider and rules engine to provide microservices. EdgeX core are implemented using Go to provide web services through multiple microservice providers. Additionally, the device proxy is implemented using Go based on the implementation template from the EdgeX foundry. The intelligent service provider is developed in Python based on Flask framework to provide microservices. In the experiment, these modules are deployed in a Raspberry Pi 4, which includes 4 GB memory to run the Ubuntu 64 bit OS smoothly. 

The IoT device is an Android application that runs on the Android Thins platform based on Raspberry Pi 3. Jetty is a Java library used for implementing the HTTP server on the IoT device to provide web services. TensorFlow lite is used for implementing the environment emulator on the IoT device to emulate the indoor environment and heater. Volley is an Android library for implanting the HTTP client. The HTTP client is used for publishing evens to the edge gateway to trigger the intelligent approach based on the rules engine.

The edge server provides the inference model through training the building user data using the Deep Neural Network (DNN) learning model on the high-performance machine. The learning model is implemented based on TensorFlow 2 framework in the Python environment. In the first step, the edge server outputs a TensorFlow DNN model, which is a folder that includes multiple files. Then, a converter is used to convert the model to a TensorFlow lite model, which is a single file with the extension tflite. Finally, we deploy the file to the edge gateway to provide intelligent services.

The detailed development environments of the IoT device, edge gateway and edge server are presented in Table 1, including the platforms, frameworks and libraries.

**Figure 8:**
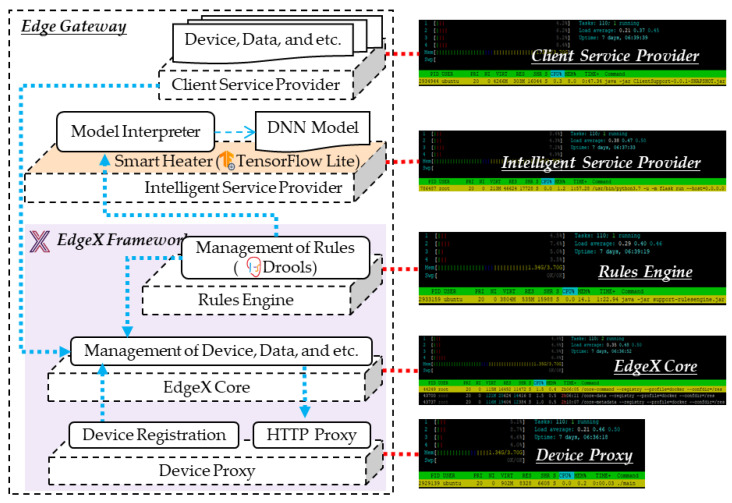
Implementation of the microservice providers run on edge gateway.

Figure 8 shows the implementation result of the proposed edge gateway, which comprises five microservice providers, including client service, intelligent service, rules engine, EdgeX core and device proxy. The providers run on the edge gateway based on Raspberry Pi 4, which can provide sufficient storage and computing ability. The client service provider provides services to edge clients for visualizing information through UIs and by forwarding the requests to the EdgeX core. The intelligent service provider includes the smart heater model, which was developed using TensorFlow Lite to provide the embedded inference approach through interpreting the DNN model. 

The rules engine, EdgeX core and device proxy are developed based on the EdgeX framework. The rules engine includes rules management that is developed using Drools to provide the rule-based service scenario. Drools is a framework that provide the rule-based approach to implement the knowledge-based systems using rules. The rules represents the knowledge to process acquired knowledge into a knowledge base that is used for reasoning to operate the corresponding actions. The rules engine can be implemented based on the if-then-else concept by a simple implementation. However, the EdgeX framework adopts the Drools framework as the default solution to implement the rules engine. Therefore, we use the Dloors framework to implement the dynamic approach for selecting the inference model to provide the intelligence in edge computing. The microservices of the intelligent service provider are triggered by the rules engine. The rules engine always detects events from the EdgeX core. The EdgeX core exposes management functions of device, data, etc. through APIs. Additionally, the provider exposes an event publishing API to receive sensing and status data from IoT devices. The device proxy registers the information of IoT devices that are deployed in the same network with the edge gateway, and forwards the request to the IoT devices. We captured the application running status using htop, which is an application monitoring software to monitor memory, processing and other additional information.

**Figure 9:**
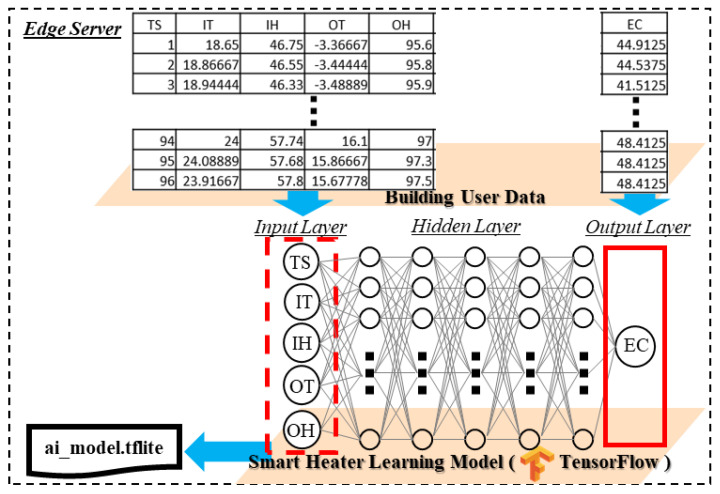
Implementation of the Deep Neural Network (DNN) model using building user data for predicting heater energy consumption.

Figure 9 shows the implementation details of the DNN-based inference model that is deployed in the intelligent service provider module to provide intelligent services. The edge server is a high-performance computer that is used for processing the building user data in the smart heater learning model to result in the DNN prediction model. The file ai_model.tflite contains the DNN prediction model that is derived from the smart heater learning model. The learning model is a DNN that is developed in TensorFlow for learning the building user data. The DNN comprises five hidden layers, and each layer has 30 nodes. The input layer has five nodes, including TS, IT, IH, OT and OH, which stand for the time sequence, indoor temperature, indoor humidity, outdoor temperature, and outdoor humidity, respectively. EC is a node in the output layer which stands for energy consumption. The learning model is trained by the building user data. TS, IT, IH, OT and OH are the input of the learning model that derives the EC. The input parameters present the environment where the user is involved. In a status of the environment, the user updates the environment to be a user-desired status using the heater through consuming the energy that is presented by EC. Therefore, we train the leaning model using input parameters TS, IT, IH, OT and OH with the output parameter EC to derive the inference model for providing a value to update the environment to be a user-desired environment. The building user data includes the time sequence, indoor temperature, indoor humidity, outdoor temperature, outdoor humidity and heater’s energy consumption. The data contain 26,208 rows that are collected from 1 January 2014 to 30 September 2014 with 15-min intervals. For training the DNN model with the building user data, the input dimension is (1, 5) and the output demension is (1, 1). The data are applied on the proposed DNN model with 3000 epochs to get a satisfying result for deploying in the intelligent service provider. However, the training process takes several minutes on the edge server; the inference model can derive the result in few microseconds (ms) in the edge gateway.

**Figure 10:**
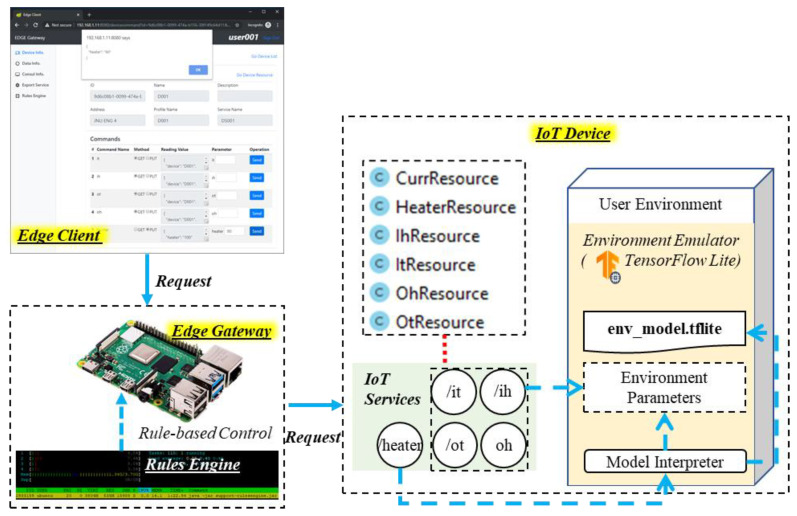
Implementation result of accessing IoT device based on rules engine and edge client.

Figure 10 shows the implementation of the IoT device. The requests to the IoT device are sent by the edge gateway through the device proxy. The implemented IoT device provides services through resource classes CuurResource, HeaterResource, IhResource, ItResource, OhResource and OtResource. On the edge of the network, the exposed IoT services are /it, /ih, /ot and /oh for sensing indoor and outdoor temperature and humidity, and /heater for actuating the heater. The IoT device includes an emulator of the user environment that was developed based on TensorFlow lite. The emulator includes a TensorFlow prediction model that predicts the updated environment parameters by the current environment with the heater energy consumption. The IoT device is requested for getting sensing and status data from the edge client, and controlling the heater based on intelligent operation through the rules engine. 

**Figure 11:**
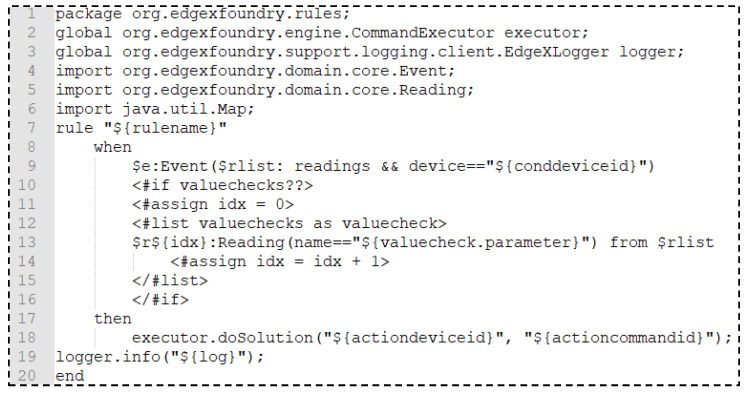
Drools template in the rules engine for creating a Drools profile.

Figure 11 shows the Drools template for creating the Drools profile. The profile is used for operating the proposed intelligent scenarios in the rules engine based on the Drools framework. Once rule information is delivered by the client service provider to the rules engine, the information is converted to a Drools file with file extension drl. The temperate defines the format of the Drools file, which includes the event filter list and objective function. An event comes with the name of the resources, which are defined in the Drools rule for triggering the objective function. The temperate creates a rule with device ID, resource name list, objective device ID and command ID. The device ID and resource name list are used for the rule condition. The objective device ID and command ID are used for operating the goal based on the rule.

**Figure 12:**
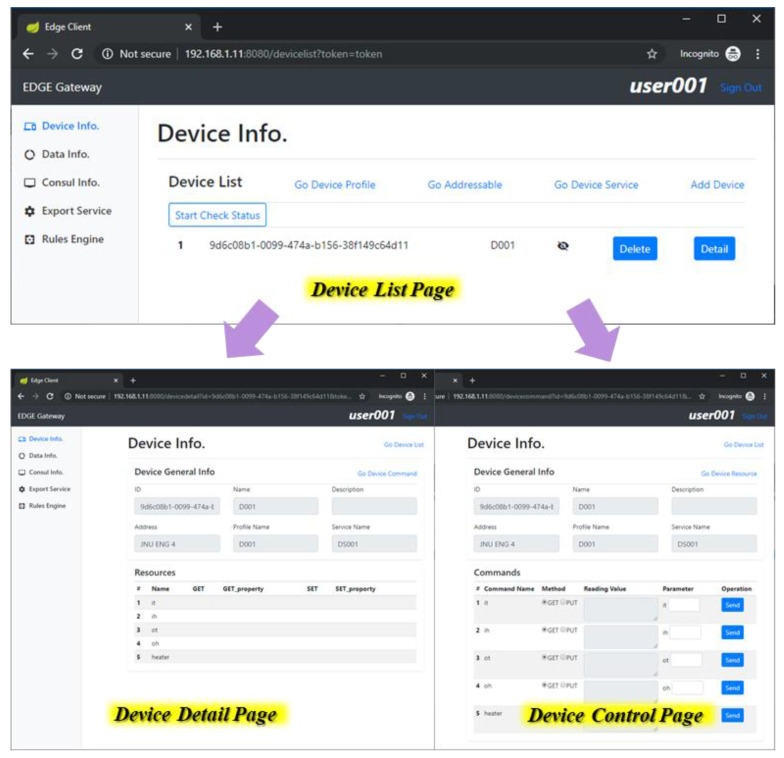
Presentation of the IoT device information on the edge client.

Figure 12 shows the implementation result of edge client that presents the information of an IoT device. The information is delivered from the EdgeX core through the Internet. For presenting the device information to users, the client shows the list of retrieved devices that are deployed in the same network with the edge gateway. Once an item is selected by a user, then the client shows detailed information of the selected device, and the page includes a link to the detail control page for sending commands to the IoT device.

**Figure 13:**
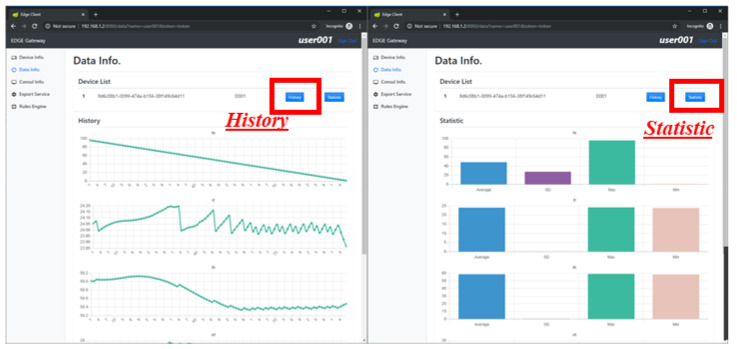
Data visualization on edge client.

Figure 13 shows the implementation result of the edge client for data visualization. The implemented IoT device provides five resources, including four sensors and one actuator. However, the IoT device sends six parameters, including a timestamp with the sensing data and actuator status. The client provides data visualization by retrieving the stored data from the edge gateway. Using the data, the client displays the maximum, minimum, average and standard deviation by deriving the statistical values. 

**Figure 14:**
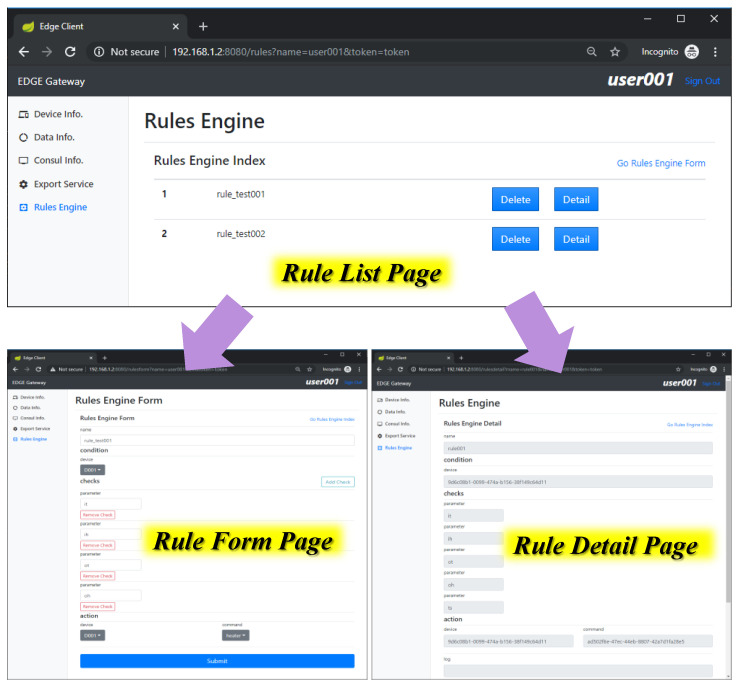
Rules management using edge client.

Figure 14 shows the implementation result of rules management in the edge client. A rule can be uploaded by a client through the edge client. The rule is deployed in the edge gateway to operate the intelligent scenario once the rule is triggered by the published event. For managing rules in the proposed edge computing, the edge client provides list, form and detail pages for retrieving and uploading rule information. The rule list page provides a list of retrieved rules that are uploaded by users through the rule form page. The rule form page provides a form for filling the information of a new rule, and through the client service provider, sends this to the rules engine, which converts the rule information to a Drools file. Detailed information of a rule is retrieved from the client service, which saves the rule information when the rule is uploaded.

## 6. Performance Evaluation

**Figure 15:**
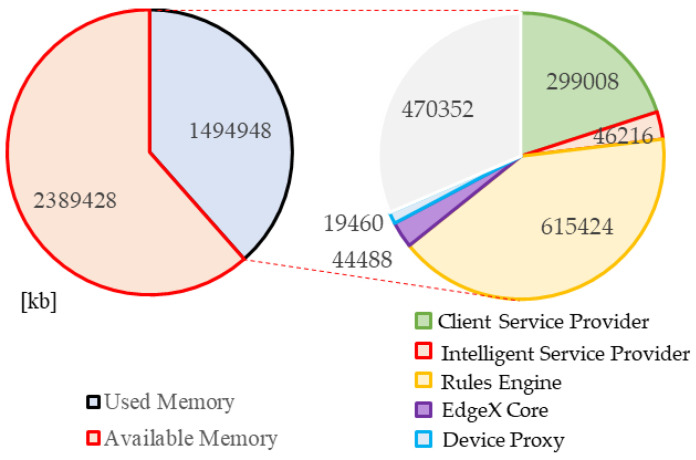
Proposed edge gateway memory usage.

For evaluating the performance of computational resources, the memory usage of the proposed edge gateway on the Raspberry Pi 4 is presented in Figure 15. The total memory of the device is 3,884,376 kb, in which the run processes take 1,499,948 kb. The device runs the proposed modules to provide the dynamic inference approach in intelligent edge computing. However, the memory ability is sufficient for running these processes. In the running processes, the edge gateway takes 1,024,596 kb, and 470,352 kb is used for other processes, including running the OS. The rules engine and client service provider modules use the most memory. Both modules are developed in Java to provide web services. Therefore, the Java applications based on Java Virtual Machine (JVM) use more memory. Increasing the rules in the rules engine does not increase the memory usage of the module. The intelligent service provider takes 46,216 kb for providing intelligent approaches based on the TensorFlow lite model. The memory usage is not increased by increasing the count of the model in the intelligent service provider. The EdgeX core runs on the Docker container, which is developed in Go to provide microservices through consuming 44,488 kb of memory. The device proxy is also developed in Go to provide services that use 19,460 kb of memory. According to the experiment, Go-based modules use less memory than Java-based modules, while they provide more functions through microservices.

**Figure 16:**
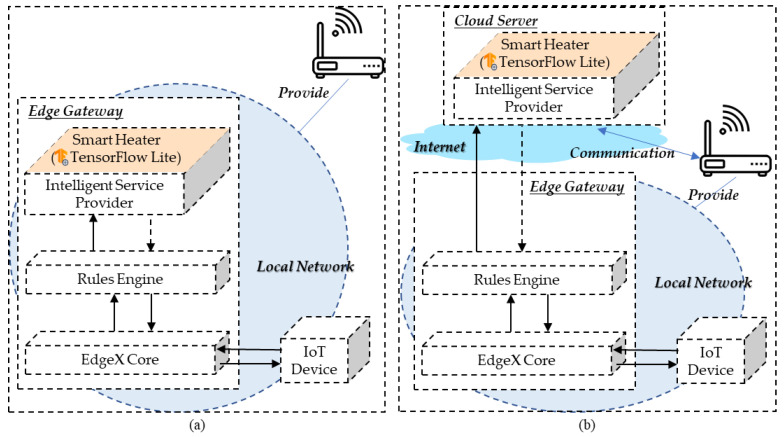
Network architecture for experimenting internal and external intelligent functions. (**a**) Internal intelligent function. (**b**) External intelligent function.

As shown in Figure 16, the experiment of the performance evaluation is performed through two types of network configurations. The proposed edge computing is configured based on deploying the intelligent function in the edge gateway, which provides the intelligent service that is performed close to the environment where the data are generated. For a comparison with the proposed approach, the external intelligent function is proposed, which is configured based on deploying the intelligent function in the cloud server. The external intelligent function provides the intelligent service from the high-performance server through the Internet. However, the inference model in the server process the request quickly based on sufficient computing ability. Nevertheless, the network delay is a potential issue in the overall latency. In the experiment with the internal intelligent function, the IoT device sends the event to the rules engine through the EdgeX core. Then, the rule is triggered and the microservice of the intelligent function is invoked by the rules engine. The delivered inference data is used for updating the environment by the edge gateway. The overall process is performed in the network edge. In the experiment with an external intelligent function, once the rule is triggered, the rules engine requests the cloud server to get the control factor, and applies the data to the environment. For providing the performance evaluation, the process delays are collected and presented through comparisons.

**Figure 17:**
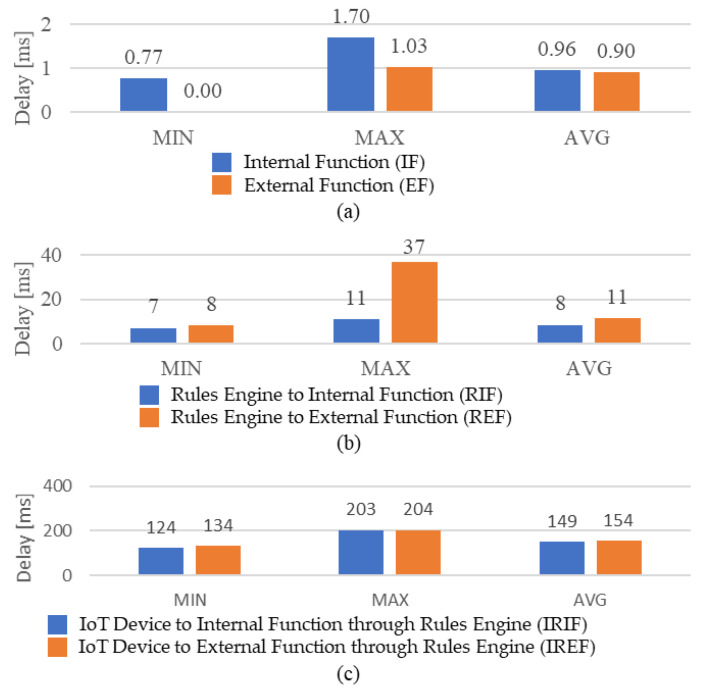
Performance of request delays for intelligent operation. (**a**) Operating intelligent function time. (**b**) Invoking intelligent function time. (**c**) Total operation time.

Figure 17 shows request delays for operating intelligent services based on the edge gateway in the network edge. For evaluating the performance of the operation latency, the comparisons between deploying the intelligent service provider on the inside and outside of the edge gateway are presented. 

Figure 17a presents the operation time of the intelligent function in the intelligent service provider module. IF means that the intelligent service provider module is deployed in the edge gateway to operate the intelligent function. EF mean thats the intelligent service provider module is deployed in the external machine. In this experiment, the external machine is a PC that includes i5-8400 CPU with SSD to provide a high-performance computing ability in a different network. Therefore, the EF presents less latency for operating the intelligent function. Nevertheless, the intelligent function operation provides small latency in the overall process. 

Figure 17b presents delays for invoking the intelligent function from the rules engine. RIF means invoking the internal intelligent function and REF means invoking the external intelligent function. The average latency shows the REF takes more time than the RIF. Although the EF takes less time for operating the intelligent function, the overall process for invoking the intelligent function through microservices takes more time because of the network delay. Figure 17c presents delays for the process, which includes event publishing of IoT device, rule activation in the rules engine, invoking the intelligent function of the intelligent service provider and sending the command to IoT device. IRIF means the experiment with the internal intelligent service provider support and IREF means the experiment with the external intelligent service provider. Through this experiment, deploying the intelligent function in the edge gateway takes less latency even when the edge gateway is deployed in a small machine with a constrained specification.

According to the experimental results, the external server machine takes less time than the edge gateway for operating the intelligent function due to its sufficient computing resources. However, edge computing enables the computational process close to the environment where the data are collected and applied. Therefore, the presented results depict that the proposed edge gateway takes less average time in the overall process, which is enabled to handle the operation of real-time intelligent scenarios in the network edge.

We applied intelligent edge computing to the building environment by predicting the energy consumption to update the environment to be the user-desired condition. Experimenting the intelligent services in a real environment requires too many resources and costs for evaluating the quality of services. Therefore, the operation of the intelligent scenario is performed in the emulated environment that is developed using deep learning models with the user data. The environment emulator includes two models, including the indoor temperature prediction model and the indoor humidity prediction model, which are used for updating the indoor temperature and humidity, respectively. The models receive the indoor temperature/humidity, outdoor temperature/humidity and energy consumption as the inputs to predict the indoor temperature or humidity. The IoT device gets the updated indoor temperature and humidity from the environment emulator and publishes this to the edge gateway to continue a rotation of the intelligent scenario. Each prediction model is trained using the same user data that are also used for the inference model in the intelligent service provider. The Mean Absolute Percentage Error (MAPE) for the temperature and humidity prediction models are, respectively, 2.65% and 4.27%, which are insufficient as compared to the real environment. Nevertheless, the evaluation results can be referred to to develop the intelligent scenario testbed.

**Figure 18:**
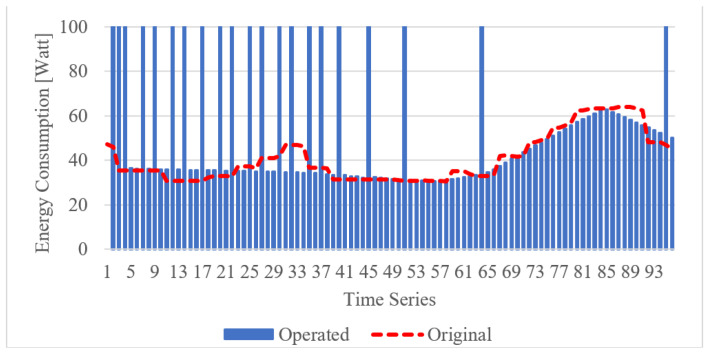
Energy consumption comparison of intelligent heater operation and original user data.

The specification of the smart heater model that depicts the output is the user-desired energy consumption for operating the heater to update the indoor temperature and humidity in the building. The model is developed based on the proposed DNN with the building user data, which are compared with the result to evaluate the performance of the prediction accuracy. Based on the model, the edge gateway applies the energy consumption on the IoT device, and the IoT device updates the temperature and humidity through the heater. Figure 18 depicts the performance of the smart heater model by comparing the operating energy consumption with the original data. The model is derived using the smart heater learning model based on DNN and building user data for providing the energy consumption from the intelligent service provider module in the edge gateway. The energy consumption result is collected 96 times. One day is separated into 96 time points, and each time point lasts 15 min. The smart heater model applies 100 watts when the indoor temperature is too low. The collected results present similar data to the original data. 

**Figure 19:**
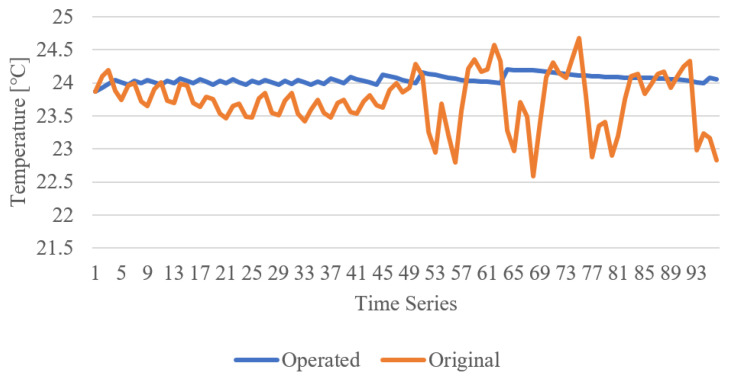
Temperature data comparison of experimental result and original user data.

Figure 19 shows the temperature data comparison between the experimental result and the original user data. The indoor temperature is updated by the heater that is operated by the edge gateway. Using the building user data, the model is developed for the indoor environment. Therefore, the temperature is similar to the original data. However, the operating temperature is kept at 24 degrees Celsius. The reason for this is that the model is configured to control the heater to always keep the indoor temperature higher than 24 degrees Celsius. The result of operating heater energy consumption illustrates that the heater sometimes consumes the maximum amount of energy.

Through the proposed experiment, we present the performance of the intelligent approach that is offloaded to the network edge. The latency performance illustrates the proposed edge computing is enabled to handle the intelligent operation immediately in the network edge. Then, the performance of the prediction results illustrates that the intelligent approaches are performed in the network edge for building environment control. 

## 7. Conclusions and Future Directions

We proposed an intelligent edge computing for building environment control through providing a dynamic inference approach using the edge gateway. The edge gateway includes the inference model to provide the intelligent service close to the environment where the sensors and actuators are deployed for collecting environmental data and updating the environment. Therefore, offloading the intelligent function to the network edge reduces the process latency for real-time control. According to the experimental results, the external server machine takes less time than the edge gateway for operating the intelligent function due to its sufficient computing resources. Nevertheless, the presented results depict that the edge gateway takes a less average time in the overall process. Moreover, the edge gateway includes rules engine to provide an intelligent service dynamically through selecting one of the multiple intelligent service providers. The microservices architecture enables deploying multiple service providers to interact with internal functions in the runtime of the edge gateway. According to the experimental results, operating the proposed microservices modules in the network edge based on the edge gateway is sufficient for providing device management, device proxy, client service, intelligent service and rules engine.

For providing the dynamic inference approach in edge computing, a rules engine and an intelligent service provider are included on the edge gateway to select and operate the intelligent model. Comparing with emerging IoT and edge computing architecture such as Open Conectivity Foundation (OCF) [50] and oneM2M [51], microservice implementation is the key to enable the dynamic inference model in the edge computing. The core specification of OCF provides the functionality of messaging, discovery, monitoring and maintenance based on the fundamental communication ability for constrained devices. Recently, the OCF core optional specification provide the rule mechanism based on the rule-specific resource that selects a function to operate based on an if-then-else structure [52]. However, the intelligent approach is not considered the base of the OCF reosurces. Different from OCF and EdgeX, the oneM2M addresses large-scale industrial solutions for logistic, factories and cities. For the implementation of oneM2M, the edge computing node is construted as a Middle Node-Common Service Entity (MN-CSE). The intelligent model can be deployed in the Application Entity (AE) on top of MN-CSE to provide intelligent services in the network edge [53]. However, the deployment of multiple intelligent models in runtime is not supported in the CES. Additionally, the rule management is not supported to operate an AE.

In the future, we will develop more inference models to deploy on the proposed edge gateway to operate multiple intelligent scenarios in the IoT network. Then, based on the multiple inference models, we can apply an optimization approach to select the optimal model. Moreover, we will develop an automatic offloading mechanism to update the inference models in the proposed edge gateway.

## Figures and Tables

**Table 1:** Development Environment.

Entity	Platform	Frameworks and Libraries
IoT Device	Raspberry Pi 3 Model B, Android Things 1.0 (Android SDK 27)	Jetty 9.1.0, Volley 1.1.0, TensorFlow-Lite 1.10.0
Edge Gateway	Raspberry Pi 4 Model B, Ubuntu 20.04 64 bit	Client Service Provider (Spring Boot 2.1.4, HTTP Client 4.5.10, Bootstrap 3.3.7, JQuery 3.4.1) Intelligent Service Provider (TensorFlow-Lite 2.1.0, Flask 1.1.2) Rules Engine (Spring Boot 2.1.4, Drools 7.11) EdgeX Core and Device Proxy (EdgeX Fuji)
Edge Server	PC, Windows 10 pro 64 bit	IoTivity 1.2/Californium CoAP/Jetty 9.1

## Data Availability

No statement.

## References

[1] Shi W., Cao J., Zhang Q., Li Y., Xu L. (2016). Edge Computing: Vision and Challenges. IEEE Internet Things J..

[2] Shi W., Dustdar S. (2016). The Promise of Edge Computing. Computer.

[3] Hong C.-H., Varghese B. (2019). Resource management in fog/edge computing: A survey on architectures, infrastructure, and algo-rithms. ACM Comput. Surv..

[4] Khan W.Z., Ahmed E., Hakak S., Yaqoob I., Ahmed A. (2019). Edge computing: A survey. Futur. Gener. Comput. Syst..

[5] Yu W., Liang F., He X., Hatcher W.G., Lu C., Lin J., Yang X. (2018). A Survey on the Edge Computing for the Internet of Things. IEEE Access.

[6] Want R., Schilit B.N., Jenson S. (2015). Enabling the Internet of Things. Computer.

[7] Jin W., Kim D. (2018). A Sleep Scheme Based on MQ Broker Using Subscribe/Publish in IoT Network. Int. J. Adv. Sci. Eng. Inf. Technol..

[8] Al-Fuqaha A.I., Guizani M., Mohammadi M., Aledhari M., Ayyash M. (2015). Internet of Things: A Survey on Enabling Technologies, Protocols, and Applications. IEEE Commun. Surv. Tutor..

[9] Salman O., Elhajj I., Kayssi A.I., Chehab A. Edge computing enabling the Internet of Things. Proceedings of the 2015 IEEE 2nd World Forum on Internet of Things (WF-IoT).

[10] Yousefpour A., Fung C., Nguyen T., Kadiyala K., Jalali F., Niakanlahiji A., Kong J., Jue J.P. (2019). All one needs to know about fog computing and related edge computing paradigms: A complete survey. J. Syst. Arch..

[11] Liu C.-F., Bennis M., Debbah M., Poor H.V. (2019). Dynamic Task Offloading and Resource Allocation for Ultra-Reliable Low-Latency Edge Computing. IEEE Trans. Commun..

[12] Morabito R., Petrolo R., Loscrí V., Mitton N. (2018). LEGIoT: A Lightweight Edge Gateway for the Internet of Things. Futur. Gener. Comput. Syst..

[13] Chen C.-H., Lin M.-Y., Liu C.-C. (2018). Edge Computing Gateway of the Industrial Internet of Things Using Multiple Collaborative Microcontrollers. IEEE Netw..

[14] Morabito R., Petrolo R., Loscrí V., Mitton N. Enabling a lightweight Edge Gateway-as-a-Service for the Internet of Things. Proceedings of the 2016 7th International Conference on the Network of the Future (NOF).

[15] Jin W., Kim D.-H. IoT device management architecture based on proxy. Proceedings of the 2017 6th International Conference on Computer Science and Network Technology (ICCSNT).

[16] Jin W., Kim D. (2019). Resource Management Based on OCF for Device Self-Registration and Status Detection in IoT Networks. Electronics.

[17] Jin W., Kim D. (2018). Development of Virtual Resource Based IoT Proxy for Bridging Heterogeneous Web Services in IoT Networks. Sensors.

[18] Jin W., Kim D. (2019). Improved Resource Directory Based on DNS Name Self-Registration for Device Transparent Access in Heterogeneous IoT Networks. IEEE Access.

[19] Zhou Z., Chen X., Li E., Zeng L., Luo K., Zhang J. (2019). Edge Intelligence: Paving the Last Mile of Artificial Intelligence with Edge Computing. Proc. IEEE.

[20] Dragoni N., Giallorenzo S., Lafuente A.L., Mazzara M., Montesi F., Mustafin R., Safina L., Mazzara M., Meyer B. (2017). Microservices: Yesterday, Today, and Tomorrow. Present and Ulterior Software Engineering.

[21] Di Francesco P., Lago P., Malavolta I. Migrating Towards Microservice Architectures: An Industrial Survey. Proceedings of the 2018 IEEE International Conference on Software Architecture (ICSA).

[22] Newman S. (2015). Building Microservices: Designing Fine-Grained Systems.

[23] Fowler M., Lewis J. (2014). Microservices.

[24] Santana C., Alencar B., Prazeres C. Microservices: A mapping study for internet of things solutions. Proceedings of the 2018 IEEE 17th International Symposium on Network Computing and Applications (NCA).

[25] LeCun Y., Bengio Y., Hinton G. (2015). Deep learning. Nature.

[26] Marquez G., Johnson B., Jafari M., Gomez M. Online Machine Learning Based Predictor for Biological Systems. Proceedings of the 2019 IEEE Symposium Series on Computational Intelligence (SSCI).

[27] Hoi S.C., Wang J., Zhao P. (2014). Libol: A library for online learning algorithms. J. Mach. Learn. Res..

[28] Edgex Foundry. https://www.edgexfoundry.org.

[29] Liu F., Tang G., Li Y., Cai Z., Zhang X., Zhou T. (2019). A Survey on Edge Computing Systems and Tools. Proc. IEEE.

[30] Zhao R., Wang X., Xia J., Fan L. (2020). Deep reinforcement learning based mobile edge computing for intelligent Internet of Things. Phys. Commun..

[31] Wang X., Han Y., Wang C., Zhao Q., Chen X., Chen M. (2019). In-Edge AI: Intelligentizing Mobile Edge Computing, Caching and Communication by Federated Learning. IEEE Netw..

[32] Mao Y., You C., Zhang J., Huang K., Letaief K.B. (2017). A Survey on Mobile Edge Computing: The Communication Perspective. IEEE Commun. Surv. Tutor..

[33] Sufyan F., Banerjee A. (2020). Computation Offloading for Distributed Mobile Edge Computing Network: A Multiobjective Approach. IEEE Access.

[34] Ceselli A., Premoli M., Secci S. Cloudlet network design optimization. Proceedings of the 2015 IFIP Networking Conference (IFIP Networking).

[35] Sanaei Z., Abolfazli S., Gani A., Buyya R. (2014). Heterogeneity in Mobile Cloud Computing: Taxonomy and Open Challenges. IEEE Commun. Surv. Tutor..

[36] Satyanarayanan M., Schuster R., Ebling M., Fettweis G., Flinck H., Joshi K., Sabnani K. (2015). An open ecosystem for mobile-cloud convergence. IEEE Commun. Mag..

[37] Satyanarayanan M., Chen Z., Ha K., Hu W., Richter W., Pillai P. Cloudlets: At the Leading Edge of Mobile-Cloud Convergence. Proceedings of the 6th International Conference on Mobile Computing, Applications and Services.

[38] Abbas N., Zhang Y., Taherkordi A., Skeie T. (2018). Mobile Edge Computing: A Survey. IEEE Internet Things J..

[39] Mach P., Becvar Z. (2017). Mobile Edge Computing: A Survey on Architecture and Computation Offloading. IEEE Commun. Surv. Tutor..

[40] Sun X., Ansari N. (2016). EdgeIoT: Mobile Edge Computing for the Internet of Things. IEEE Commun. Mag..

[41] Dolui K., Datta S.K. Comparison of edge computing implementations: Fog computing, cloudlet and mobile edge computing. Proceedings of the 2017 Global Internet of Things Summit (GIoTS).

[42] Yu S., Wang X., Langar R. Computation offloading for mobile edge computing: A deep learning approach. Proceedings of the 2017 IEEE 28th Annual International Symposium on Personal, Indoor, and Mobile Radio Communications (PIMRC).

[43] Eom H., Juste P.S., Figueiredo R., Tickoo O., Illikkal R., Iyer R. Machine Learning-Based Runtime Scheduler for Mobile Offloading Framework. Proceedings of the 2013 IEEE/ACM 6th International Conference on Utility and Cloud Computing.

[44] Qiao G., Leng S., Zhang K., He Y. (2018). Collaborative Task Offloading in Vehicular Edge Multi-Access Networks. IEEE Commun. Mag..

[45] Xu J., Chen L., Ren S. (2017). Online Learning for Offloading and Autoscaling in Energy Harvesting Mobile Edge Computing. IEEE Trans. Cogn. Commun. Netw..

[46] Crutcher A., Koch C., Coleman K., Patman J., Esposito F., Calyam P. Hyperprofile-Based Computation Offloading for Mobile Edge Networks. Proceedings of the 2017 IEEE 14th International Conference on Mobile Ad Hoc and Sensor Systems (MASS).

[47] Kwak J., Kim Y., Lee J., Chong S. (2015). DREAM: Dynamic Resource and Task Allocation for Energy Minimization in Mobile Cloud Systems. IEEE J. Sel. Areas Commun..

[48] Zhang W., Zhao D., Xu L., Li Z., Gong W., Zhou J. Distributed embedded deep learning based real-time video processing. Proceedings of the 2016 IEEE International Conference on Systems, Man, and Cybernetics (SMC).

[49] Blanco-Filgueira B., García-Lesta D., Fernández-Sanjurjo M., Brea V.M., López P. (2019). Deep learning-based multiple object visual tracking on embedded system for IOT and mobile edge computing applications. IEEE Internet Things J..

[50] Park S. OCF: A New Open IoT Consortium. Proceedings of the 2017 31st International Conference on Advanced Information Networking and Applications Workshops (WAINA).

[51] Swetina J., Lu G., Jacobs P., Ennesser F., Song J. (2014). Toward a standardized common M2M service layer platform: Introduction to oneM2M. IEEE Wirel. Commun..

[52] OCF Core Optioanl Specification. https://openconnectivity.org/specs/OCF_Core_Optional_Specification_v2.2.1.pdf.

[53] Cai K.L., Lin F.J. Distributed Artificial Intelligence Enabled by oneM2M and Fog Networking. Proceedings of the 2018 IEEE Conference on Standards for Communications and Networking (CSCN).

